# Changes in Metabolic Profile of Rice Leaves Induced by Humic Acids

**DOI:** 10.3390/plants11233261

**Published:** 2022-11-27

**Authors:** Natália Aguiar Canellas, Fábio Lopes Olivares, Rakiely Martins da Silva, Luciano Pasqualoto Canellas

**Affiliations:** Núcleo de Desenvolvimento de Insumos Biológicos para a Agricultura (NUDIBA), Universidade Estadual do Norte Fluminense Darcy Ribeiro (UENF), Campos dos Goytacazes, Rio de Janeiro 28013-602, Brazil

**Keywords:** metabolomics, primary and secondary metabolism, GC-TOF MS, biofertiliser

## Abstract

The use of humic substances in agriculture as a biostimulant emerged as one of the promising methods to promote sustainable production. Different molecular, biochemical, and physiological processes are triggered, resulting in nutrient efficiency use and protection against abiotic stress. Understanding plant changes promoted by humic substances is essential for innovative and tailored biostimulation technologies. Cell metabolites are the final target of the response chain, and the metabolomic approach can be helpful in unveiling pathways related to plant response. This study aimed to evaluate a global metabolic alteration of rice leaves induced by humic acids (HA) applied in a hydroponics system. Using ^1^H NMR and GC-TOF/MS analysis, we observed a significant decrease in all main metabolites classes in leaves treated with HA, including lipids, organic acids, amino acids, and carbohydrates. Metabolites in higher concentrations in HA-treated plants are candidates as markers of HA bioactivity, including amino acids, intermediates of tricarboxylic acid cycle, and lipids, and aromatic compounds related to plant-stress response.

## 1. Introduction

There is a growing interest in the agricultural use of humic substances (HS). Commercial humic products are mainly obtained from geochemical sediments, such as coals and peats. However, they can also be isolated from renewable sources of stabilized organic matter, such as compost and vermicompost, allowing the farmer’s autonomy. HS are better depictured as complex mixtures of thousands of different small molecules taking a supramolecular organization when in aqueous suspension [1]. The presence of key functional groups might trigger positive physiological responses when supplied to plants at very low dosages [2]. The rising success of biostimulation by HS has been based on two main aspects: Their recognized effects on ion uptake and nutrient use efficiency [3] and plant protection against abiotic stress [4]. Direct application of HS on crops allows for maintaining productivity levels with a significant reduction in the use of inorganic fertilizers [5,6]. HS also can be used in the plant acclimatization process protecting from abiotic stress as a beneficial agent inducing downstream effects on hormonal signaling pathways and plant development [7,8,9].

Understanding how plants perceive and respond to HS applications is essential for designing and developing biostimulation technology aimed at specific applications. For example, the effect of HS on plant growth depends on the source, dose, content of bioactive molecules, and mode of HS application [2]. In addition, changes in metabolism can help assess the effect of HS on plants.

Cell metabolites are the primary targets of physiological action, but studies with the metabolomic approach in plants treated with HS are relatively scarce. A previous metabolomic survey showed that humic acids (HA) changed global metabolic distribution with a significant decrease in amino acid concentration [10]. However, target analysis has already shown an increase in some specific amino acid synthesis in plants treated with HS [11]. In addition, HA increased the levels of 40 compounds, and these included metabolites linked to the stress response (shikimic, caffeic, hydroxycinnamic acids, putrescine, behenic acid, quinoline xylulose, galactose, lactose proline, oxyproline, and valeric acid) and cellular growth (adenine and adenosine derivatives, ribose, ribonic acid, and citric acid) [10]. More recently, it was also observed that during nutrient starvation, maize treated with humic biostimulant significantly impacted pathways involved in stress-alleviating mechanisms [12].

Furthermore, the application of HS from compost showed a positive effect on the total extraction yield of steam distillation of Basil leaves. At the same time, it did not significantly change the composition of essential oils, a valuable bioactive secondary metabolite of aromatic plants [13]. A similar effect was observed in maize roots treated with HA that showed no changes in cell metabolite composition, modifying only their amount in the root tissues [14]. 

Mapping global changes in metabolism can help understand the plant-HA interactions. However, the complexity of soil as a multiphase and multicomponent system may hamper a comprehensive outlining of the humic-plant interaction mechanism of applied biostimulants on rhizosphere environments. Moreover, the soil-plant relationship is not easy to disentangle the specific priming effect of humic molecules acting as a bioactive mediator in the combination of microbial bioeffectors [7,10,14]. Therefore, this study aimed to evaluate the alterations in rice leaves metabolomic induced by HA isolated from vermicompost cattle manure applied in a hydroponics system.

## 2. Results

### 2.1. HA Characteristics

**Figure 1:**
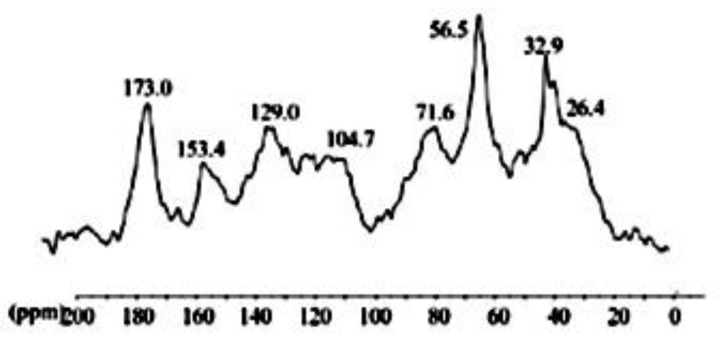
CPMAS-^13^C-NMR spectrum of humic acids isolated from vermicompost.

The ^13^C CPMAS NMR spectrum of HA isolated from vermicompost (Figure 1) was characterized by the predominance of alkyl-C interval and O-alkyl-C components, representing 25% and 40% of the total area, respectively. The former region of NMR spectra (0–45 ppm) includes the alkyl-C functions assigned to the presence of aliphatic chains of various lipid compounds [15]. The sharp band at 32.9 ppm and the shoulder around 26.4 ppm are related to the large abundance of methylene groups belonging to linear compounds with a different structural arrangement, such as microbial fatty acids, plant waxes, and biopolyesters [16,17]. The intense resonance centered at 56.5 ppm may combine the methoxyl substituent (OCH_3_) on the aromatic rings of lignin components and the C–N bonds in amino acid moieties [16]. The different peaks in the 60–110 ppm chemical shift are the typical resonances of O-alkyl-C of polysaccharide chains such as cellulose and hemicellulose of plant tissues [15]. The less evident carbon nucleus at 64 ppm is followed by the broad coalescence around 71.6 ppm composed by the spectral overlay of carbon 2, 3, and 5 in the pyranoside structure. The de-shielded signal shifted at 104.7 ppm is derived from the presence of di-O-alkyl anomeric carbon. The resonances shown along the aryl-C region (110–140 ppm) originate from both un-substituted and C-substituted phenyl carbon of lignin monomers of guaiacyl and syringyl units as well as to condensed aromatic moieties, while the following phenolic aromatic group (140–160 ppm) highlighted the presence of O substituted ring carbons in lignin molecules. The lignin compounds are typical components of manure-based composts and vermicompost, deriving from the not assimilated woody fraction of fodder crops [16]. Finally, the broadband at 173 ppm indicated the inclusion of carbonyl groups of various compounds, such as aliphatic acids, amino acid moieties, side groups of pectin derivatives, etc.

### 2.2. Changes in Metabolites Fingerprint Profile

**Figure 2:**
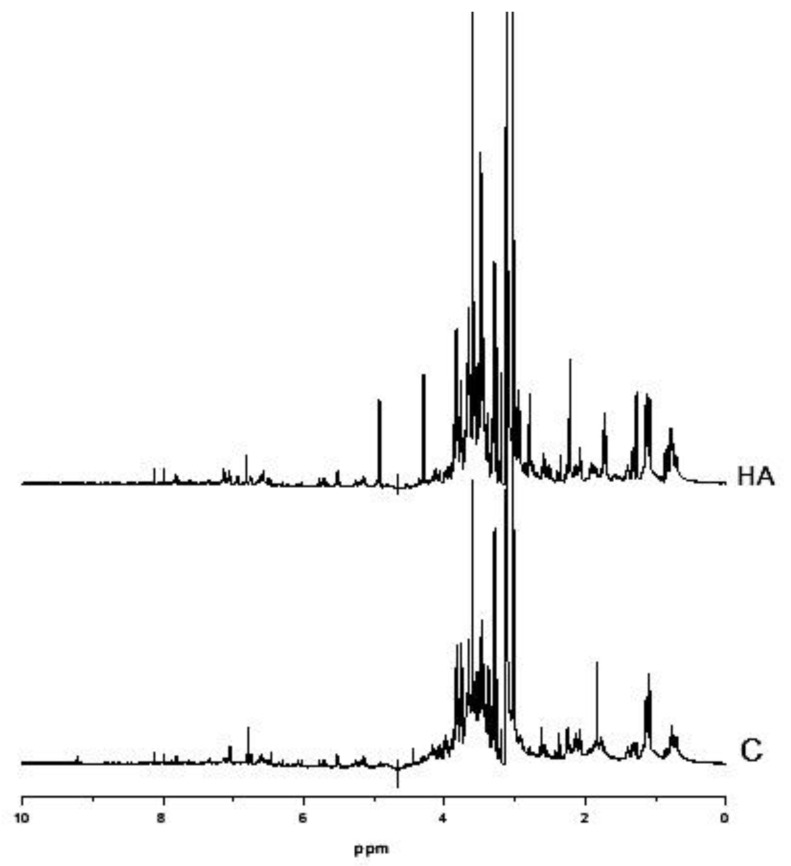
^1^ H NMR spectra of rice leaf extracts from seedlings treated with humic acids (HA) or not (C).

**Figure 3:**
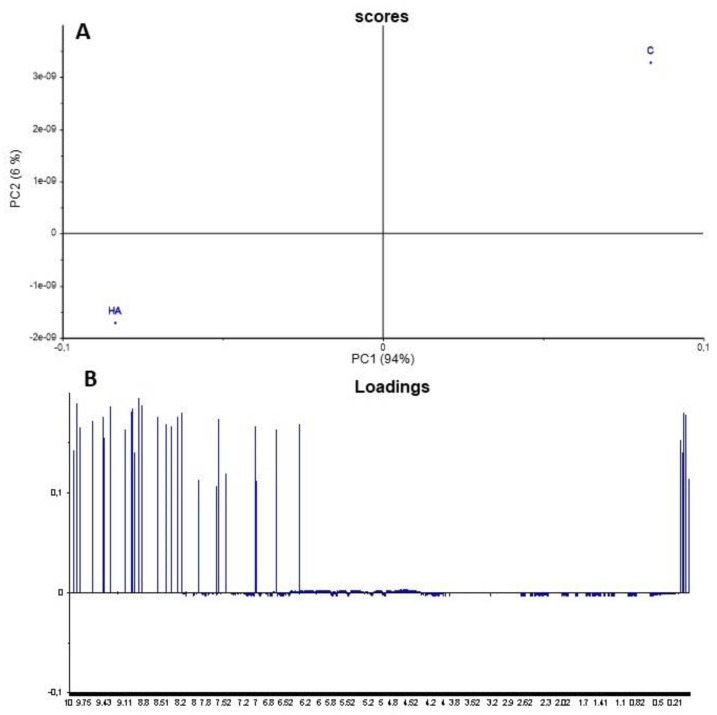
PCA scores (**A**) and loadings (**B**) show a good separation between leaf tissues of rice seedlings treated with humic acids (HA) concerning control plants (C).

The HA applied in hydroponics resulted in a significant increase of 36% in fresh leaves weight concerning control plants (0.71 ± 0.082 g plant^−1^ vs. 0.52 ± 0.045 g plant^−1^, *n* = 3 F test *p* < 0.001). The growth promotion was accompanied by changes in metabolic fingerprinting measured by ^1^H NMR, a powerful technique to compare overall metabolic compositions. The spectra of rice leaf extracts are shown in Figure 2. The complexity of molecular composition and the main differences associated with the metabolic fingerprints can be unveiled and visualized using a PCA approach (Figure 3).

The first two PCs comprised 100% of the variance. HA treatment clearly distinguishes them concerning control plants. PC1 captured 94% of the total variance, while PC2 explained 6% of the total variance. PC1 showed (Figure 3B) that control rice had a low content of protons bonded to *sp^2^*-hybridized carbons, like aromatic structures, with signals centered at 6.6 ppm and aliphatic groups with a resonance at 1.07 ppm, which were related to the rice control. 

PC2 (Figure 3) distinguished treated from untreated plants, showing high levels of aromatic and carbohydrate species that allowed the separation of the treatment. The PCA allowed the quick identification of treatment effects in rice seedlings (Figure 3). 

### 2.3. Changes in Metabolites Composition

**Figure 4:**
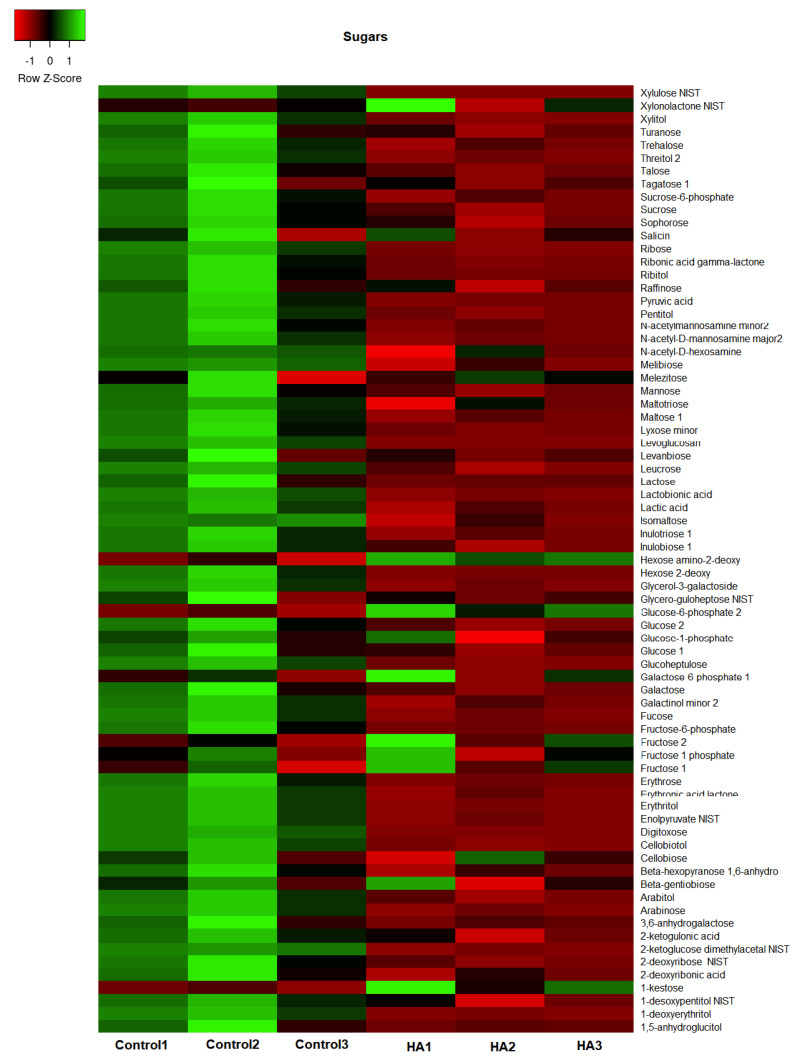
The concentration map of carbohydrates (heat map of scaled concentrations) by plants treated with humic acids (HA) or not (Control).

**Figure 5:**
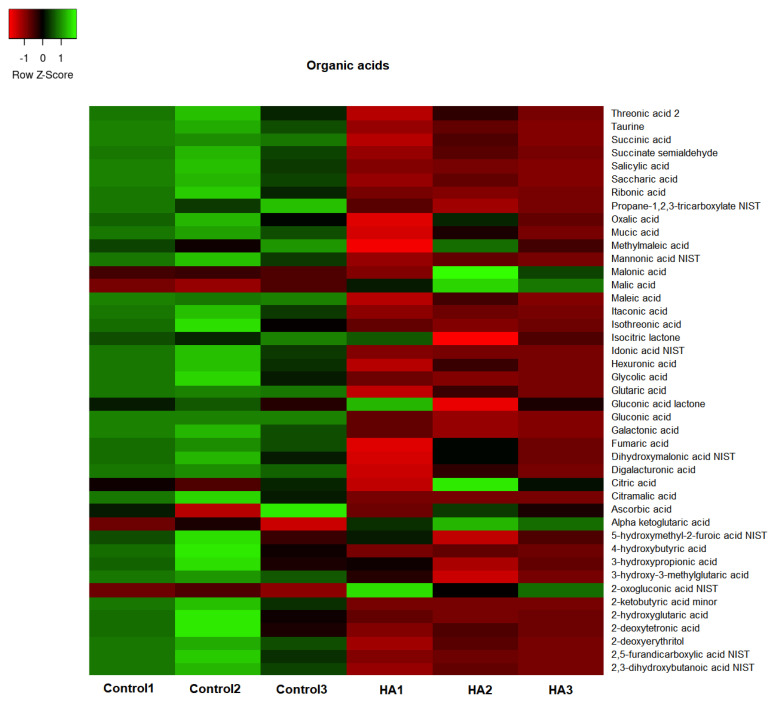
The concentration map of organic acids (heat map of scaled concentrations) by plants treated with humic acids (HA) or not (Control).

**Figure 6:**
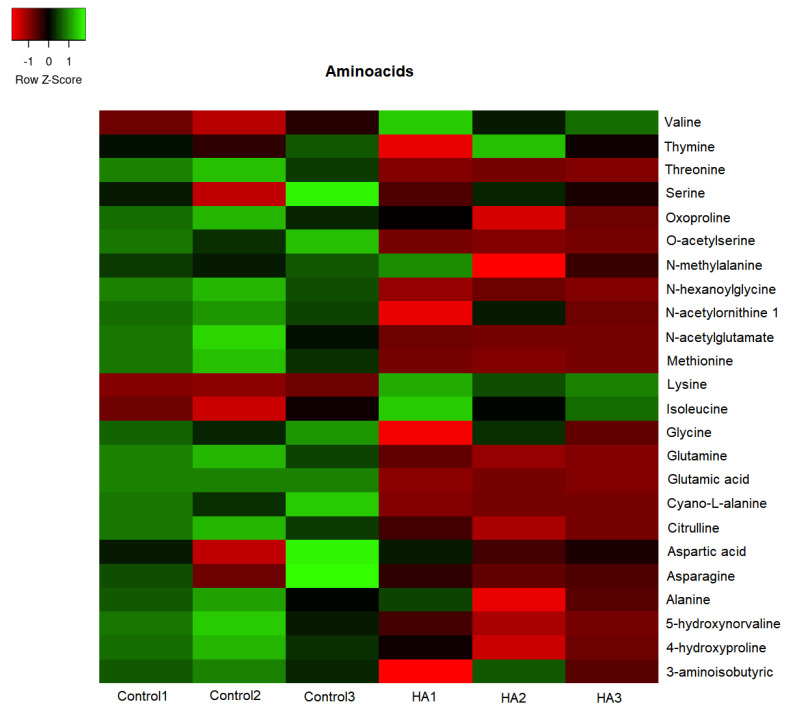
The concentration map of amino acids (heat map of scaled concentrations) by plants treated with humic acids (HA) or not (Control).

**Figure 7:**
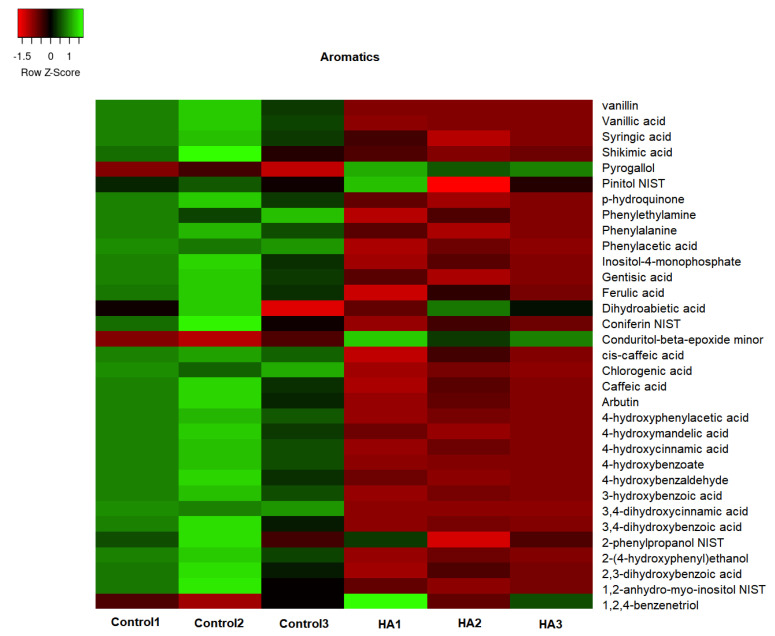
The concentration map of aromatic compounds (heat map of scaled concentrations) by plants treated with humic acids (HA) or not (Control).

**Figure 8:**
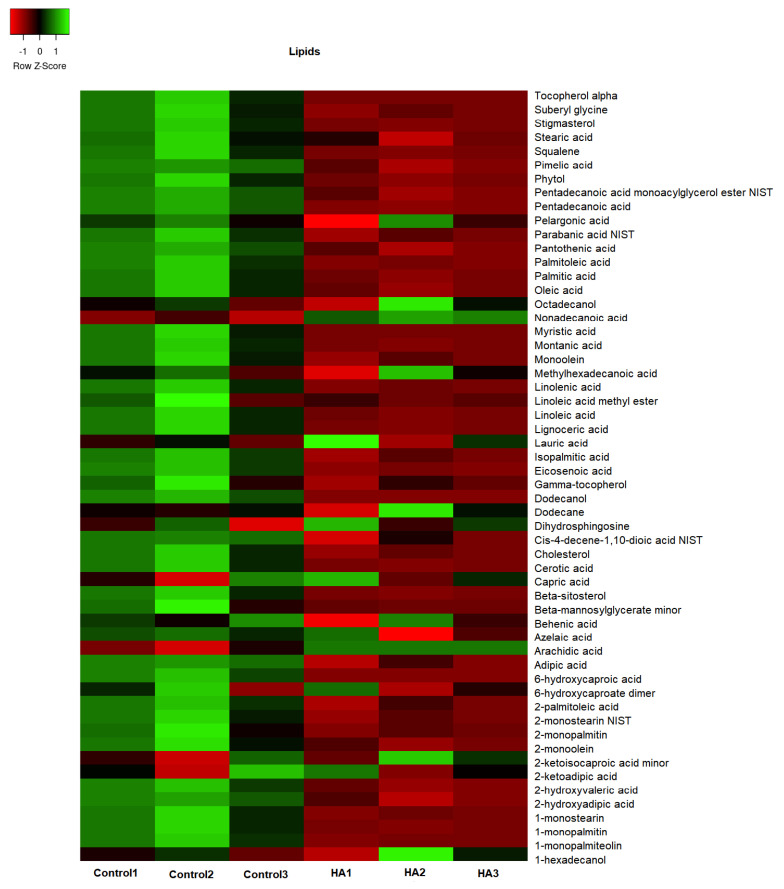
The concentration map of lipids (heat map of scaled concentrations) by plants treated with humic acids (HA) or not (Control).

The GC/TOF-MS led to the identification of 307 compounds present in the leaf extracts of rice seedlings. Figure 4, Figure 5, Figure 6 and Figure 7 present the heatmap of the metabolic levels based on the mass fragmentations patterns. After the F test (control vs. treated), we found 30 compounds in significantly high concentrations in treated plants compared to controls. These compounds are shown in Table 1 and are candidate biomarkers of the biostimulant’s action. In total, 27 of 30 compounds originate from the primary metabolism, including three amino acids (isoleucine, lysine, and valine) and five organic acids from the tricarboxylic acid cycle (citric, malic, maleic, and oxogluconic and alfa ketoglutaric acid). On the other hand, lipids were the most representative metabolites in HA-treating at a significantly higher level (Figure 8 and Table 1). Octadecane, octadecanol, hexadecanol, dodecane, azelaic, arachidic acid, and dihydrophengosine were the main lipids found in greater concentrations in treated seedlings. In addition, the phenylpropanoid pathway was significantly induced, and it was possible to observe an increase in pyrogallol and its benzenetriol derivative and cyclic polyols (conduritol derivative).

## 3. Discussion

HA isolated from vermicompost (Figure 1) showed standard chemical features usually associated with high biological activity such as methoxyl, unsubstituted aromatics, phenols, and carboxylic groups indicated by the 56 and 130 to 150 and 174 ppm chemical shift in ^13^C-CP/MAS NMR spectrum [15,16,17]. In fact, the application of HA in hydroponic systems promoted a significant alteration of the metabolic fingerprint of leaf extracts, clearly highlighted by ^1^H NMR (Figure 2). The growth promotion of humic products has been related to their impact on several molecular, biochemical, and physiological processes. Since the final target is the cell metabolites, the growth acceleration resulted in an overall decrease of cell metabolites compared with control plants (Figure 4, Figure 5, Figure 6, Figure 7 and Figure 8). However, 30 compounds were found in significantly high levels in plants treated with HA concerning control (Table 1). They are a candidate to express the effect of HA on plant metabolism since it is operationally easier to follow a metabolite when it is present in a higher concentration. The discussion will focus on the role of these compounds in plants.

Three, rather than five, sugars were found in high levels linked to fructose metabolism. One of fructose’s primary biological functions is acting as an alternative metabolite in providing energy, especially when glucose is insufficient while the metabolic energy demand is high [18]. It can enter the glycolysis path and produce intermediates for cellular respiration. Hexoses and glucose-6-P are the primary cell fuels provided by glycolysis and the pentose phosphate pathway.

Oxogluconic acids are a product of glucose oxidation. The four organic acids found in larger significant concentrations in HA-treated plants, i.e., malic, malonic, citric, and alpha-ketoglutaric acid, are produced in the tricarboxylic acid (TCA) cycle (Table 1). These organic acids are then taken into the mitochondria and subsequently interconverted to produce energy, reducing power and carbon structures. Nardi et al. [19] previously observed an induced stimulation of HS on the activity of different enzymes of TCA in maize plants. Furthermore, oxogluconic, maleic, and citric acids were detected at an enhanced rate in sugarcane crop leaves following the addition of HA isolated from vermicompost, which was thus conceived as candidates for a metabolic target of humic product activity [10]. The pivotal roles of the over-synthesized TCA-related compounds include photosynthesis, photorespiration, reductant transport, and the maintenance of photosynthetic redox balance and nitrogen metabolism [20].

Conversely, a decreasing amount of most amino acids was revealed by rice leaves extracts after adding HA, compared to control plants (Figure 6). However, three amino acids were found in high levels in the leaf tissues of rice seedlings treated with HA (Table 1). The essential amino acid lysine and the derived isoleucine are synthesized via a pathway starting with aspartate, leading to the formation of threonine and methionine [21]. The biochemical sequence involves enzymes in the plastids and homoserine kinase (EC 2.7.1.39), which catalyzes the formation of O-phosphohomoserine from homoserine and leads to the formation of either threonine or methionine [22]. The last improved amino acid, valine, together with leucine and isoleucine, contains unique branches collectively called branched amino acids. *TOR* (Target of Rapamycin) and *SnRK1* (sucrose non-fermenting-1-related protein kinase) are two crucial regulatory hubs in plant growth activated by HA [8,23], and both are regulated by valine concentration [24]. Previously, Vaccaro and colleagues [11] reported that enzyme activity involved in plant-N assimilation (GS and NADH-GOGAT system) was regulated by exogenous HA concentration. Some free amino acids in leaves were found in high levels, including aspartate, isoleucine, and lysine.

The application of HA in rice plants also affected lipids metabolism (Figure 8, Table 1). Lipids have essential structural functions in plant cell membranes, are a highly energetic carbon source for cells, and can act as cell signal messengers. These compounds are chemically defined by their low-aqueous solubility, and this broad definition includes molecules from primary and secondary plant metabolism. We identified nine lipid compounds more significantly expressed in leaf tissues of HA-treated plants (Figure 8, Table 1). Most of these lipid components found at high levels are related to plant signaling events associated with stress. For example, azelaic acid was used as a plant priming agent to induce a resistance response against a pathogen accumulating salicylic acid (SA), a known defense signal upon infection [25]. Arachidonic acid also changes the expression of JA and SA pathway genes in plants modulating stress-responsive transcriptional networks [26,27]. The two long-chain fatty alcohols (dodecanol and octadecanol) increased by HA application (Table 1) are fundamental metabolic intermediates for nutrient homeostasis and plant adaptation to adverse conditions [28]. Sphinganine or dihydrosphingosine (d18:0, DHS), one of the most abundant free sphingoid long chain base in plants, also act as signaling molecules during biotic and abiotic stresses [29].

Accumulation of polyamines and tyramine and the activities of their biosynthetic enzymes confers plant tolerance to drought or salt stress [30], and spermidine and tyramine were found in high levels in HA-plant treated (Table 1). Cytosine and methylcytosine were also found in high concentrations in plants treated with HA and are determinant building segments for cellular code and essential for cell growth and proliferation. Finally, phenolic compounds help to regulate various physiological functions in plants during growth and development and are also involved in plant defense mechanisms [31]. The biosynthesis and accumulation of phenolic compounds during stress are regulated by phenylalanine ammonia-lyase (PAL) activity. This study reported strong regulation of PAL by HA [32], and five phenolic compounds were found in high concentrations (Table 1). Pyrogallol and its derivative (benzenetriol) are one of the most soluble phenols effective against the biotic stress in plant cells [33].

Taken together, the metabolic analysis expresses a significant acceleration of the plant growth showing a general decrease of the compounds at the same time that several compounds’ markers of anti-stress response were found at a high level.

## 4. Materials and Methods

### 4.1. Humic Acids Extraction (HA)

HA was extracted and purified as reported elsewhere [15]. Briefly, 10 volumes of 0.5 mol L^−1^ NaOH were mixed with one volume of earthworm compost under an N_2_ atmosphere and shaken overnight. After 12 h, the suspension was centrifuged at 5000× *g* and acidified to a pH of 1.5 using 6 mol L^−1^ HCl. The HAs were solubilized in 0.5 mol L^−1^ NaOH and precipitated three times. The sample was repeatedly washed with water until a negative test against AgNO_3_. Next, the HA was titrated to a pH of 7.0 with 0.1 mol L^−1^ KOH and then dialyzed against deionized water using a 1 kD cutoff membrane (Thomas Scientific, Swedesboro, NJ, USA) before they were lyophilized.

### 4.2. HA Characterisation

CP-MAS ^13^C NMR–The solid-state Nuclear Magnetic Resonance (^13^C CPMAS NMR) spectra of isolated humic acids were recorded on a Bruker AV-300 equipped with a 4 mm wide-bore MAS probe, with the following acquisition parameter: 13,000 Hz of rotor spin rate; 2 s of recycling time; 1H-power for CP 92.16 W: 1H 90° pulse 2.85 μs; ^13^C power for CP 1504 W; 1 ms of contact time; 30 ms of acquisition time; 4000 scans. Samples were packed in 4 mm zirconium rotors with Kel-F caps. The Fourier transform was performed with a 4k data point and an exponential apodization of 100 Hz line broadening. For the interpretation of ^13^C-CPMAS-NMR spectra, the overall chemical shift range is split into the following main regions: Alkyl-C (0–45 ppm); methoxyl-C and N-alkyl-C (45–60 ppm); O-alkyl-C (60–110 ppm); unsubstituted and alkyl-substituted aromatic-C (110–145 ppm); O-substituted aromatic-C (145–160 ppm); carboxyl- and carbonyl- C (160–200 ppm)

### 4.3. Plant Assay

Rice seeds (*Oryza sativa* L. BR-IRGA 409) were surface sterilized by being soaked in 0.5% NaClO for 30 min, rinsed with water, and soaked in water for 6 h. Then, the seeds were sown on wet filter paper and germinated in the dark at 28 °C. Four days after germination, six rice seedlings with root lengths of approximately 2.0 cm were transferred into 0.5 L vessels filled with a solution containing 2 mM CaCl_2_ (control) or supplemented with HA (60 mg carbon L ^-1^). A minimal medium (2 mM CaCl_2_) was used to avoid any interference from nutrients that may act synergistically with humic matter during plant growth and development. The leaves were collected after 5 days of treatment. Rice seedlings were placed in a plant growth cabinet with a photoperiod of 10 h of light and 14 h of darkness, a light intensity of 120 μmol m ^−2^ s ^−1^, and temperatures of 25 °C (night) and 28 °C (day). The control plants were grown only in a minimal medium without HA.

Rice leaves were collected under controlled conditions, ground in the N_2_ homogenizer and stored at −80 °C. Metabolites were extracted using 20 mg of fresh weight sample, adding 1 mL of pre-chilled extraction solution (80:20 *v*/*v* solvent mixture of methanol/water). The samples were vortex for 10s and shaken for 6 min, followed by centrifugation for 10 min at 10,000× *g*. We remove the supernatant in two 500 μL portions, saving one as a backup. One part was dried and submitted to derivatization.

### 4.4. Metabolic Fingerprint Analysis

^1^H NMR spectroscopy was conducted at 25 ± 1 °C using a 400 MHz Avance magnet (Bruker Biospin, Rheinstetten, Germany) equipped with a 5-mm Bruker Broadband Inverse (Bruker Biospin) probe working at the ^1^H frequency of 400 MHz. Samples were prepared by dissolving 10 mg of leaf extract in 1 mL of deuterated water (99.8% D_2_O/H_2_O, Armar Chemicals). ^1^H NMR spectra were acquired by applying an on-resonance presaturation of water signal (2 s of presaturation at 54–65 dB for power level attenuation) and setting 16 k data points, four dummy scans, a spectral width of 16 ppm (6410.3 Hz) and 128 scans. Spectra were processed using Mestre-C software (v.4.8.6.0, Cambridgesoft, Cambridge, MA, USA). In particular, FIDs were transformed by applying a line broadening of 3 Hz. The ^1^H NMR spectral dataset was auto-scaled, and a PCA was performed using the software package Unscrambler X 10.2 (Camo Inc., Oslo, Norway). The dataset consisted of a matrix in which each row represented the spectral average of three leaf extracts of rice. The model validation was carried out using full cross-validation, and the difference in the variance between the calibration and validation models was less than 5%. 

### 4.5. Gas Chromatography-Time of Fly Mass Spectrometry (GC–TOF/MS)

Derivatization was performed as described previously [34]. In summary, 2 μL of a C8-C30 FAME mixture was used to convert retention times to retention index (RI). Carbonyl groups were protected by 10 μL of a solution of 20 mg mL^−1^ methoxyamine in pyridine at 30 °C for 90 min. Ninety microliters of MSTFA and 1% TMCS was added for trimethylsilylation of acidic protons at 37 °C for 30 min. After derivatization, 0.5 μL samples were injected in randomized sequence into a Gerstel cold injection system (Gerstel, Muehlheim, Germany) and Agilent 7890A gas chromatograph (Santa Clara, CA, USA) in splitless mode. The Leco ChromaTOF software controlled the system versus 2.32 (St. Joseph, MI, USA). A 30 m long, 0.25 mm i.d. RTX 5Sil-MS column with 0.25 mL 5% diphenyl/95% dimethyl polysiloxane film and additional 10 m integrated guard column was used (Restek, Bellefonte, PA, USA). The injection temperature was 230 °C, and the interface was set to 280 °C. Helium flow was 1 mL min^−1^. After a 5 min solvent delay time at 50 °C, the oven temperature was increased at 20 °C min^−1^ to 330 °C, 5 min isocratic, cool down to 50 °C and an additional 5 min delay afterwards. Liners were exchanged automatically for every 10 samples. A time-of-flight mass spectrometer (Leco Pegasus IV) was operated at a transfer line temperature of 280 °C, and the ion source was adjusted at 250 °C and −70 V electron impact ionization. Mass spectra were acquired at mass resolving power R = 600 from m/z 85 to 500 at 17 spectra s^−1^. The results files were exported to a data server with absolute spectra intensities and further processed by a filtering algorithm implemented in the metabolomics Bin-Base database [35]. Quantification was reported as peak height using the unique ion as the default unless a different quantification ion was manually set in the BinBase administration software Bellerophon. Metabolites were unambiguously assigned by the Bin-Base identifier numbers, using retention index and mass spectrum as the two most essential identification criteria. All database entries in BinBase were matched against the Fiehn mass spectral library of 1200 authentic metabolite spectra and the NIST05 commercial library [36]. A quantification report table was produced for all database entries that were positively detected in more than 50% of the samples of a study design class as defined in the SetupX database [37]. The concentration map (heat map of the scaled concentration) was built by using the heat mapper program of software R version 4.0.3 (Development Core Team 2020), using the heat mapper package.

## 5. Conclusions

The direct use of HA from recycled biomasses on plants can be included as innovative technology to enhance the sustainability of agricultural production systems by preserving soil fertility and minimizing the adverse environmental impact of current farming practices. HA-biostimulants remodel rice plant metabolism towards growth promotion via the activation and enhancement of physiological events for improved plant development, resulting in decreased cell metabolite concentration. However, fewer compounds were found in high concentrations, especially those linked with the potentiation of defenses, and are candidates for metabolic markers of HA bioactivity. Based on the variable composition of composted biomasses, an essential requirement is represented by the comprehensive understanding of structural activity relationship with advanced metabolomic approaches. Therefore, scientific research is an unavoidable support to delineate an innovative and tailored application of humic biostimulants as sustainable and self-learning farming practices.

## Figures and Tables

**Table 1:** Cell metabolic compounds identified by GC-TOF/MS were found in significantly larger concentrations by F test (*p* < 0.001) in leaf tissues of rice seedlings treated with humic acids isolated from vermicompost.

Sugars	Organic Acids	Amino Acids	N-Compounds	Aromatics	Lipids
hexose	Citric	Valine	Cytosin	benzenetriol	Azelaic acid
Fructose	Malic	Isoleucine	Hydroxylamine	Dihydroxy benzenic acid	Dodecanol
Fructose 1-P	Malonic acid	Lysine	Inosine	Pyrogallol	hexadecanol
Fructose 6-P	Oxogluconic		Methylcitosine		Dihydrophingosine
Glucose-6-P	Alpha ketoglutaric		Spermidine		Arachidic acid
			Tyramine		Nonedecanoic acid
					Octadecanol

## Data Availability

Not applicable.
